# The Text Mining Technique Applied to the Analysis of Health Interventions to Combat Congenital Syphilis in Brazil: The Case of the “Syphilis No!” Project

**DOI:** 10.3389/fpubh.2022.855680

**Published:** 2022-03-30

**Authors:** Marcella A. da Rocha, Marquiony M. dos Santos, Raphael S. Fontes, Andréa S. P. de Melo, Aliete Cunha-Oliveira, Angélica E. Miranda, Carlos A. P. de Oliveira, Hugo Gonçalo Oliveira, Cristine M. G. Gusmão, Thaísa G. F. M. S. Lima, Rafael Pinto, Daniele M. S. Barros, Ricardo A. de M. Valentim

**Affiliations:** ^1^Laboratory of Technological Innovation in Health, Federal University of Rio Grande do Norte, Natal, Brazil; ^2^Health Sciences Research Unit: Nursing (UICISA:E), Nursing School of Coimbra (ESEnfC), Coimbra, Portugal; ^3^Postgraduate Program in Infectious Diseases, Federal University of Espírito Santo, Vitoria, Brazil; ^4^Multidisciplinary Department of Human Development With Technologies, State University of Rio de Janeiro, Rio de Janeiro, Brazil; ^5^Centre for Informatics and Systems of the University of Coimbra, Department of Informatics Engineering, University of Coimbra, Coimbra, Portugal; ^6^Department of Biomedical Engineering, Federal University of Pernambuco (UFPE), Recife, Brazil; ^7^Ministry of Health, Brasília, Brazil

**Keywords:** text mining, public health, congenital syphilis, content analysis, “no syphilis” project

## Abstract

Congenital syphilis (CS) remains a threat to public health worldwide, especially in developing countries. To mitigate the impacts of the CS epidemic, the Brazilian government has developed a national intervention project called “Syphilis No.” Thus, among its range of actions is the production of thousands of writings featuring the experiences of research and intervention supporters (RIS) of the project, called field researchers. In addition, this large volume of base data was subjected to analysis through data mining, which may contribute to better strategies for combating syphilis. Natural language processing is a form of knowledge extraction. First, the database extracted from the “LUES Platform” with 4,874 documents between 2018 and 2020 was employed. This was followed by text preprocessing, selecting texts referring to the field researchers' reports for analysis. Finally, for analyzing the documents, N-grams extraction (*N* = 2,3,4) was performed. The combination of the TF-IDF metric with the BoW algorithm was applied to assess terms' importance and frequency and text clustering. In total, 1019 field activity reports were mined. Word extraction from the text mining method set out the following guiding axioms from the bigrams: “confronting syphilis in primary health care;” “investigation committee for congenital syphilis in the territory;” “municipal plan for monitoring and investigating syphilis cases through health surveillance;” “women's healthcare networks for syphilis in pregnant;” “diagnosis and treatment with a focus on rapid testing.” Text mining may serve public health research subjects when used in parallel with the conventional content analysis method. The computational method extracted intervention activities from field researchers, also providing inferences on how the strategies of the “Syphilis No” Project influenced the decrease in congenital syphilis cases in the territory.

## 1. Introduction

Syphilis is a sexually transmitted disease (STD) that surfaced in Europe in the late 15th-century (1, 2). While this condition is preventable, curable, and usually treatable with penicillin injections, it remains a global health security threat. In addition, syphilis is the second major cause of adverse pregnancy outcomes, including neonatal death (3–6). That fact raises the syphilis epidemic as a neglected problem that poses considerable challenges to many health systems.

Although there is an effective therapy to combat the infectious agent, Treponema pallidum, measures to control and prevent the disease require that health care systems integrate many levels of care. That includes planning and deploying preventive and therapeutic interventions for all stages and types of syphilis in different population groups. In sum, the infection is relatively simple to treat if diagnosed at an early stage but difficult to control among populations (3, 4).

In 2016, the World Health Organization (WHO) launched an initiative to eliminate congenital syphilis (CS). Its goal was that CS case counts would not exceed 50 cases per 100,000 live births in 80% of countries (7). However, the overall rate was 473 cases per 100,000 live births in this very period (8). In Brazil, rates of reported CS outnumber those of other nations, with 880 cases per 100,000 live births in 2017, thus representing an Average Annual Percent Change (AAPC) of 60.38% (8).

Confronted by the growing syphilis epidemic, the Ministry of Health (MoH) and governing bodies of Brazil's Unified Health System (SUS) developed a national project for implementing and integrating a syphilis response into healthcare networks (9). The "Syphilis No!" Project (SNP), as it was titled, represented the instrument with which the MoH implemented a public health response to reduce syphilis in pregnant women (SIP) and eliminate congenital syphilis in Brazil. It has been in progress since 2018 and is built on two cornerstones: (1) actions with a universal scope and (2) specific actions in priority areas. Specific actions were developed in 100 municipalities the Ministry of Health considered a priority due to the high burden of CS (9, 10).

A team of field researchers carried out the specific actions in line with the five thematic areas of the project (Surveillance, Management and Governance, Integral Care, Education, and Communication) to qualify the local response on syphilis. It seems clear that describing the work of RIS in the priority areas renders substantive material to support analyses on the impact of interventions and their associations as a tool to induce health policy responses to syphilis.

The “LUES Platform” was one of the resources of the project. It was developed and used to monitor field researchers' activities. In such a digital platform, the researchers entered reports or individual texts related to their activities in the territory. Nonetheless, well beyond the need to interpret data on the project's associations in the territory, it is clear that research approaches that interpret textual production (e.g., content or thematic analysis) pose challenges. This is closely related to the scarcity of consolidated methods for using and processing large volumes of text.

Theorists like Bardin (2011) have discussed computers' relevance and practicality to deepen content analysis techniques, as long as researchers prepare unambiguous instructions. From this standpoint, content analysis automation can occur in varying degrees (total or partial). That implies direct consequences on the analytic practice, such as increased speed, increased rigor in research setup, and the potential for complex data manipulation, and so on (11).

An additional challenge for research done with sheer volumes of text data is to objectively interpret structures that aid in understanding the impact of work included on reports available on digital platforms, or at least to attempt to do so. Thus, it would take years to analyze such types of data through conventional manual methods. To address this issue, there are software-related strategies for text analysis that are typically applied in the healthcare field (12).

However, the currently available software is insufficient for monitoring strategic intervention activities. This mostly happens because they produce limited information that does not consider textual modeling (13). Such software limits the ability of researchers to handle the results generated safely. In addition, there are several difficulties in interpreting the word processing in such programs (13).

As the use of Artificial Intelligence continues to expand across many healthcare fields, machine learning algorithms (MLA) for big data analysis and modeling started to emerge (14, 15). In this vein, the text mining technique has the potential to provide consistency in the interpretation of text monitoring strategies, as well as a reliable treatment for the rendering of word form n-grams (16). However, the strategy of using text mining techniques to identify the main intervention activities in large national projects, such as the “Syphilis No!” Project, has not been found within the current literature.

Incorporating text and data mining (TDM) and the content analysis method into research involving large volumes of data has the potential to support evaluative studies (17). Further, it can supplement indicators that assess the effectiveness of work processes and their performance, which will further assist public health strategies toward interventions dealing with challenges similar to congenital syphilis. Therefore, with the aid of a set of algorithms – which make up a computational method applied to text mining – it is possible to identify which intervention activities developed by the RIS may have spurred public health policy strategies in the territory. In other words, strategies that point to a significant reduction in CS cases.

This article considers that the “Syphilis No!” Project has been contributing to syphilis cases reduction in Brazil. Our assumption is that field researchers' practices demonstrate the link between the goals of the project and the management of syphilis in the territory. Furthermore, their activities could have potentially influenced the indicators of syphilis in priority municipalities.

Hence, this article's scope is to analyze the connections amid the goals of the “Syphilis No!” Project and the content presented by the field researchers by using computational methods that apply text mining algorithms–in addition to discussing the role of such connections as health public policy drivers.

### 1.1. Related Works

We consider it adequate to briefly quote some studies that use computational methods, whether NLP methods or techniques based on Machine Learning (ML) to differentiate our use of Natural Language Processing. The analysis aimed to identify applications in several areas related to health. El Kah and colleagues studied a large volume of data produced through the implementation of an electronic patient record system (18). To efficiently handle these complex data, researchers employed NLP to extract patterns and elements of interest. By Valentim et al. (19) studied a similar Stochastic Petri Net Model describing the relationship between reported maternal and congenital syphilis cases in Brazilaspect; however, in a different field of application, i.e., the Virtual Learning Environment of the Brazilian Health System (AVASUS). In that example, the goal was to identify the writing style for authorship recognition using NLP methods. In (20), it was possible to find some methodological similarities regarding the work developed by Rocha et al. (21). Machine Learning methods enabled an optimized and more accurate analysis of text content and regular expressions. In our case, applying neural networks also proved to be a feasible way to work with NLP and TDM techniques.

Accordingly, the Clinical Record Interactive Search (CRIS) is another work applied in healthcare by means of content analysis. This work is developed within the context of large volumes of clinical data provided by the South London and Maudsley Trust. Its focus was to search for highly relevant elements in unstructured text, and for this purpose, NLP proved to be particularly important. Such practice prompted access to relevant results about the clinical status of patients.

Thus, it was observed that nearly 7,500 people affected by schizophrenia presented with a clinical picture of negative symptoms, which often leads to impaired cognitive ability, disordered thinking, and lack of concentration. Thus, NLP approaches were proposed, which enabled obtaining a more assertive context from the documents (22).

Regarding electronic patient records, the high volume of these records always stands out, especially when health information systems are centralized. Therefore, computational methods applied to content analysis can significantly contribute to this field, especially in patients' clinical histories studies.

Some studies focus on ubiquitous data acquisition, such as the work developed by Javed et al. (23). Such a study was underpinned by a generic, intelligent, autonomous, and collaborative Internet of Things (IoT) architecture. The proposed model is based on smartphones and machine learning. One of its most potent contributions is sharing health-related information, which can be used in comparative analysis. Furthermore, the ubiquitous model proposed in this study fosters the centralization and production of large volumes of data. Therefore, computational methods, especially regarding content analysis, can be of much value to measure, both quali- or quantitatively, the behavior of users of collaborative health settings, where the main objective is to evaluate the daily activities of these users.

These research efforts were applied in different contexts in the health field, yet they all used computational methods, whether NLP methods or techniques based on Machine Learning (ML). The studies in question approached aspects of patient care and were applied in clinical settings. However, computational methods were used in different contexts in all the studies, which indicates a fertile ground for scientific research and diverse applications. It is noteworthy that none of the studies listed used Natural Language Processing or Machine Learning to evaluate the impact of public health policies, which is one of the main contributions of this article.

## 2. Materials and Methods

According to Bardin (11), content analysis is a set of methodological tools that, being continuously perfected, can be applied to varying types of text. These tools underscore the development of data categories and ratify how relevant it is to understand the significance of the context in which analyzed items have been applied (11).

The primary function of content analysis is to unravel the critic about a particular discourse (11). It can be done qualitatively or quantitatively. In the first approach, the analysis is performed both systematically and analytically. Hence, a researcher reviews themes or categories of analysis through conceptualization, data collection, analysis, and interpretation. In the second, the search is oriented for the quantification of content as to predetermined categories; and it is conducted in a systematic and replicable manner (11, 24).

In both approaches, the goal is to infer knowledge about the semantics of the prevailing discourse in the data or its message. In this study, we adopted a qualitative-quantitative approach. This stems from the fact that such an approach mixes TDM techniques and a conceptualization of the extracted discourse–with a theoretical basis that seeks to understand the intervention activities–from the perspective of public health in the territory. In this context, the theoretical basis that served as a contributing factor in determining this research's development steps was inspired by Bardin (11), and it is characterized into three main stages, namely: (i) pre-analysis; (ii) exploring the materials: text mining; and (iii) analysis of Obtained Results: Inference and Interpretation, implemented in the Python programming language version 3.7.

These steps were essential to the development of this work. Given the volume of texts produced by the field researchers, the text mining method and subsequent content analysis were the most suitable approaches to study our research subject. Moreover, text mining was necessary to analyze the formation of the most relevant word statistically' and subsequently form logical semantic sentences based on content analysis.

### 2.1. Pre-analysis

Pre-analysis is the process in which the corpus is organized so it becomes operational and by which preliminary ideas are systematized. In this step, we carried out three tasks: (i) skimming, for an overall comprehension of the documents that would be collected; (ii) documents selection, which consisted in determining which ones would be analyzed; and (iii) formulating the hypotheses and objectives of the research to be developed (11).

In the pre-analysis, it was necessary to grasp (i) the central features that characterize the “Syphilis No!” Project in order to propose the subject of the present study; (ii) the ethical issues related to the research; and (iii) the features of the corpus (texts) for analysis in the next step—exploring the materials.

### 2.2. Features of the “Syphilis No!” Project

The SNP was developed by the Brazilian government through the Ministry of Health (MH). Over the last ten years, it has been the leading syphilis-related public health intervention in Brazil (8, 10, 25).

Within the breadth of strategies to combat syphilis, this study focuses on the institutional support network composed of field researchers who worked in priority municipalities in the regions of Brazil. Such a support network was assembled with the primary purpose of strengthening the “nexus between the Project and the health services managers in the territory,” “to coordinate programmatic actions agreed upon within governance bodies of SUS with local plans” and to offer support for a “timely response to syphilis within the healthcare networks” (26).

The project relied upon 52 research and intervention supporters distributed across 72 of the 100 priority areas in all five Brazilian regions (26). RIS worked alongside health services managers to find the best strategies to confront syphilis. The following determinations guided them: evaluating the health plans and programs of local health departments; strengthening committees for investigating mother-to-child transmission (MTCT); strengthening strategic information systems for health surveillance and improving case reporting, laboratory follow-up, and closure of syphilis cases, as well as operationalizing the line of care for adult syphilis and congenitally exposed infants (10).

All activities developed by RIS were monitored by supervisors who used a digital management system, that is, the “LUES Platform.” Through the platform, the researchers produced and submitted individual monthly reports in free text format (not systematized). Researchers produced and submitted individual monthly reports in free-form (not systematized) through the platform. These reports were based on the on-site experience and endeavors, as well as on the results of interactions between the project's actions and interventions in the public health agenda agreed upon in the inter-federative context of Brazil's SUS. It is particularly noteworthy that throughout the operationalization of the SNP, the Ministry of Health, responsible for implementing health policies in response to syphilis in Brazil, assigned the role of coordinating the syphilis policy and its set of actions at the local level to each RIS (8).

### 2.3. Research Ethics Issues

The database of texts related to the SNP interventions used in this article is in the public domain. In this manner, secondary data are used without any sensitive identification of the participants or public actors. Data were gathered after the extraction and anonymization of the documents released in the repository of the “LUES Platform.” Of note, this data can be easily accessed by any researcher at the following link: http://vigilanciasaude.ufrn.br/files/anonymous_reports.csv.

### 2.4. Exploring the Materials

Each field researcher individually wrote the reports selected for content analysis. Texts could be written in a free format (not systematized), meaning that each supporter developed their own reports featuring an account of what was done and accomplished. Therefore, the project did not provide any template for RIS to follow. Of note, the MoH entrusted each field researcher with a set of actions to be carried out on a monthly basis during the SNP. Actions were aligned with the project's goals and the public health agenda.

Since reports featured free-form content and the health terms structures are identifiable—using the analytical method, by two, three, or more words—the TDM process was fully automated by text mining algorithms using N-grams (see “Words and data extraction” for detailed information). By applying the analytical method, we observed it was not enough to identify any intervention action taken by the field researchers that only one term. However, from two to five words, identification could be achieved.

Text mining may be defined as the automated discovery of hitherto unknown knowledge utilizing an unstructured database (14, 15). A keystone of this method is linking combined information to form new facts or new hypotheses to be explored.

The following actions were carried out for implementing the text mining technique in this research: (i) detailing the Database; (ii) pre-processing; (iii) Words and Data Extraction; and (iv) N-grams Extraction. Each of the activities is subsequently described in detail.

### 2.5. Detailing the Database

The database was extracted from the “LUES Platform.” The texts collected refer to accounts about technical visits and meetings; and progress reports concerning work and research plans, thus totaling 4,874 text file documents–this sum corresponded to 3.86 Gigabytes of digitally stored files. Each document has nearly 740 words. The time frame for compiling the corpus was from May 2018 to December 2020.

In order to build the database, those documents underwent the process of anonymization and identification. Afterward, they were indexed according to the information provided in Table 1.

**Table 1:** Document Indexing Data.

**Variable**	**Description**
Identifier	Document identification number
Month	Month the document was created
Year	Year the document was created
State	Brazilian State where the document was created
Region	Brazilian Region where the document was created

Each entry represents a document that has been collected. This document catalog has been saved in a comma-separated values (CSV) format, a text file separated by commas (27). The file is available for download at the following link: http://vigilanciasaude.ufrn.br/files/anonymous_reports.csv.

### 2.6. Pre-processing

Pre-processing consisted of 4 tasks: (i) Document Standardization; (ii) Text Normalization; (iii) Removal of Duplicate Documents; and (iv) Text Correction. The objective of the first task was to standardize the text database by saving the files separately. Then, each text document was processed and renamed according to the following structure: fileA_B.C, where:

- A = activity number of the Research and Intervention Supporter in the platform;
- B = identification number of the document, generated by the “LUES Platform;”
- C = file extension.

For instance, a file could be defined as: **file2923_22305.docx**.

**Figure 1:**
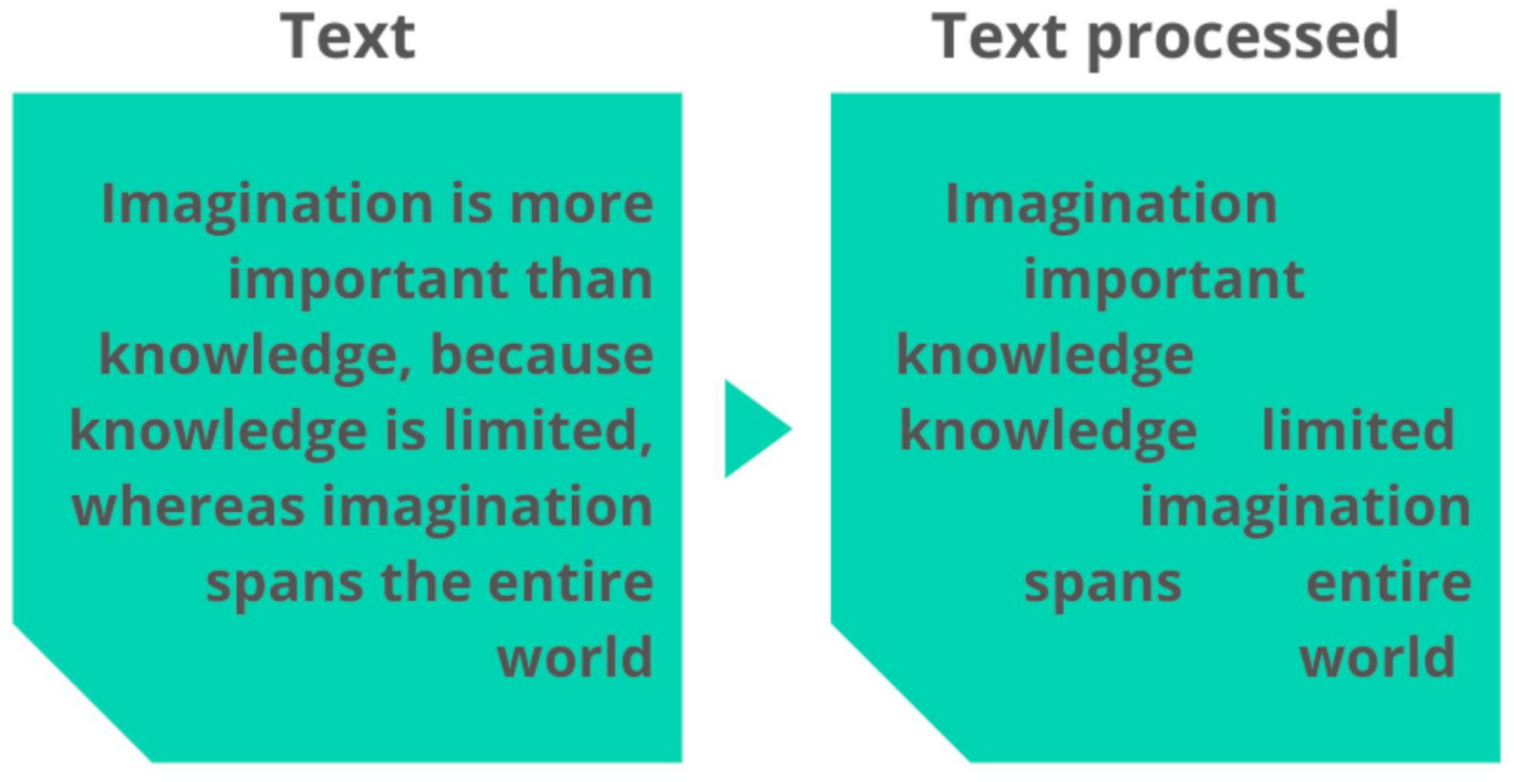
Example of word processing.

The second task was text normalization. The purpose was to (a) standardize the text in lowercase; (b) delete extra spaces between characters; (c) remove any special characters, symbols, duplicate words in a row; and (d) remove accents and numbers. After that, a stop words dictionary was used to remove non-significant aspects of the language components. Since the text reports were written in Brazilian Portuguese, this was the language of the dictionary. Hence, it includes 204 unique words that are considered undesirable for meaningful analysis, such as: “de,” “a,” “o,” “que,” “e,” “é,” “do,” “da,” “em,” “um,” and so forth (28). An example of word processing is shown in Figure 1.

The third task consisted in removing the duplicate documents. Finally, the fourth one was to make the necessary text corrections, such as grammar error correction and adding missing spaces in some sentences.

### 2.7. Words and Data Extraction

For capturing words relevance within the text documents, it was necessary to highlight those sentences that came up often and were related to the topic in the texts. Therefore, not attaching any weight to those found in all files–sentences that were obvious in the portrayed context.

To obtain a representation of the topic addressed in the documents and to extract the sentences, the N-gram approach was applied, with *N* = 2,3,4 across the entire group of texts, as well as grouped n-grams using the content analysis method. Finally, the total number of field researchers and reports per region of Brazil was calculated.

### 2.8. N-grams Extraction: Bigrams, Trigrams, and Quadrigrams

In this step, automatic extractions of keywords formed by a sequence of two words were made. The algorithm also deduced it as automatically relevant in the text documents of Intervention Researchers, the bigrams (N-gram with *N* = 2) (29, 30).

The first step toward extracting the bigrams was to obtain a list of the ordered words from the text (tokens) through the bag-of-words (BoW) approach. Subsequently, the BoW model was converted into a two-word count matrix. Moreover, the Term Frequency—Inverse Document Frequency (TF-IDF) metric was applied (31, 32). Such a technique statistically measures the importance of a word within a text in relation to other pieces within the same database. The value of the word's importance increases proportionally as the total occurrence of that word in the text increases. Thus, the value is compensated by the frequency of that word in the database. That helps discriminate the occurrence of some words that are pretty common but unimportant within the text.

There are two steps to calculating the TF-IDF: (1) separately calculating the TF and then the IDF and (2) multiplying the two parts to find the final value. First, Equation (1) calculates the TF, which measures the probability of a word w occurring in a text *t*.


(1) $$TF_{pt}=\frac{n_{pt}}{\sum_{k}n_{kt}}$$

Where *n*_*pt*_ is the number of times the word *w* occurs in the text *t*; in the denominator, it is the sum of the frequency of all the words in the text. Subsequently, Equation (2) calculates a word's overall relevance across all the texts in the database, IDF.


(2) $$\begin{array}{l} IDF_{p}=\log\frac{\left|T\right|}{\left|t_{p}:n_{pt}\neq 0\right|} \end{array}$$

Where |*T*| indicates the total number of texts in the database and the number of texts in which the word occurs at least a single time. The bigrams were also explored and clustered by region of Brazil. What is more, the same procedure was applied to other extracted n-grams.

When extracting the trigrams (N-gram with *N* =3), the keywords were formed by a sequence of three words. The same procedure was used for the bigram. However, with the counting matrix being obtained with the formation of three words.

For the extraction of the quadrigrams (N-gram with N=4), the keywords were obtained by a four-word sequence, using the same method as for the bigram, whereby the count matrix was attained by forming four words.

N-grams were extracted and grouped by Brazilian region and year. We performed that step based on identifying the structures derived from the levels of health care, syphilis rapid response actions, and other predominant activities observed in reports. Then, the proportion of terms was calculated considering both groups (region and year), as follows:


(3) $$\begin{array}{l} Proportion_{termo}=\frac{\sum N-gram\;per\;report}{number\;of\;reports\;per\;region\;and\;year} \end{array}$$

### 2.9. Analysis of Obtained Results: Inference and Interpretation

It is understood that the text production of the field researchers encompasses important content to emphasize the connections of the “Syphilis No” Project in the territory, especially when it comes to CS. In that case, it is about the healthcare field. More specifically, it is about the local response to syphilis and the data, or inputs, on the field researchers' observations and analyses during the project's operationalization. Moreover, in this study, “Syphilis No” is regarded as a public health intervention, based on the public health programs approach by Zulmira Hartz, who analyzes such interventions as actions that favor “adaptive behaviors across distinct human fields or activities” (33).

In the content analysis method, Bardin affirms, deductions are proposed from the significant results and according to the research goals (11). In this research, after the text mining with the formation of the most important bigrams, trigrams, and quadrigrams, a logical sequence was used for the conceptual clustering, depending on the different processes for actions for confronting syphilis that the field researchers have employed. In the conceptual clustering of the terms, the idea was to identify the structures of healthcare levels, programmatic actions in response to syphilis within healthcare networks, and the most prevalent stages identified in the reports produced.

## 3. Results

**Figure 2:**
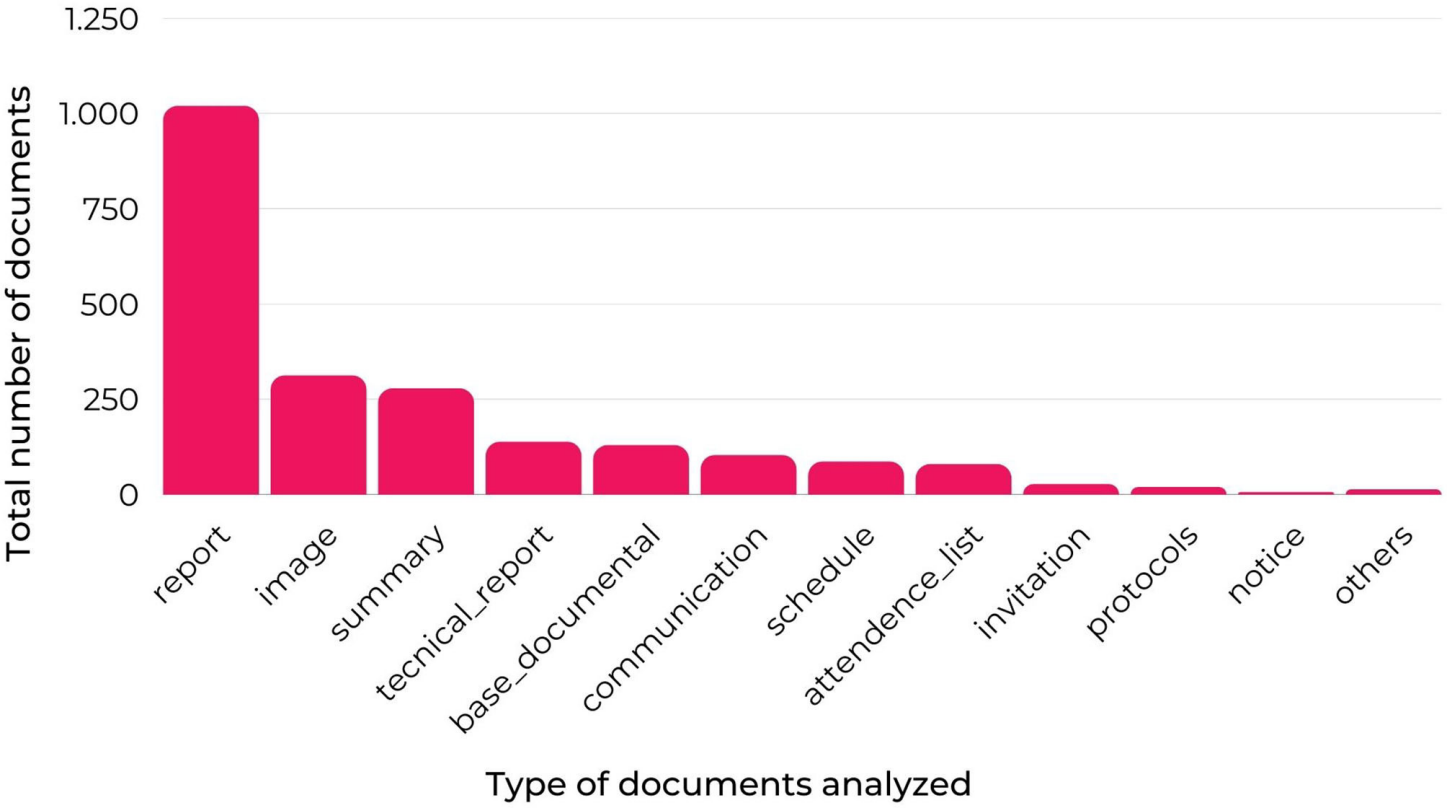
Types of files in the “LUES Platform”.

Of the 4874 files inserted into the “LUES Platform” in the 2018–2020 period by field researchers, which after pre-processing, totaled 2164, the most are reports, thus totaling 1,019 files, as depicted in Figure 2. In addition, other kinds of textual productions were entered into the system, such as images (%), abstracts (%), technical reports (%), communications (%), events or meeting schedules (%), attendance lists (%), and more types of text files (articles, epidemiological bulletins, etc.).

**Figure 3:**
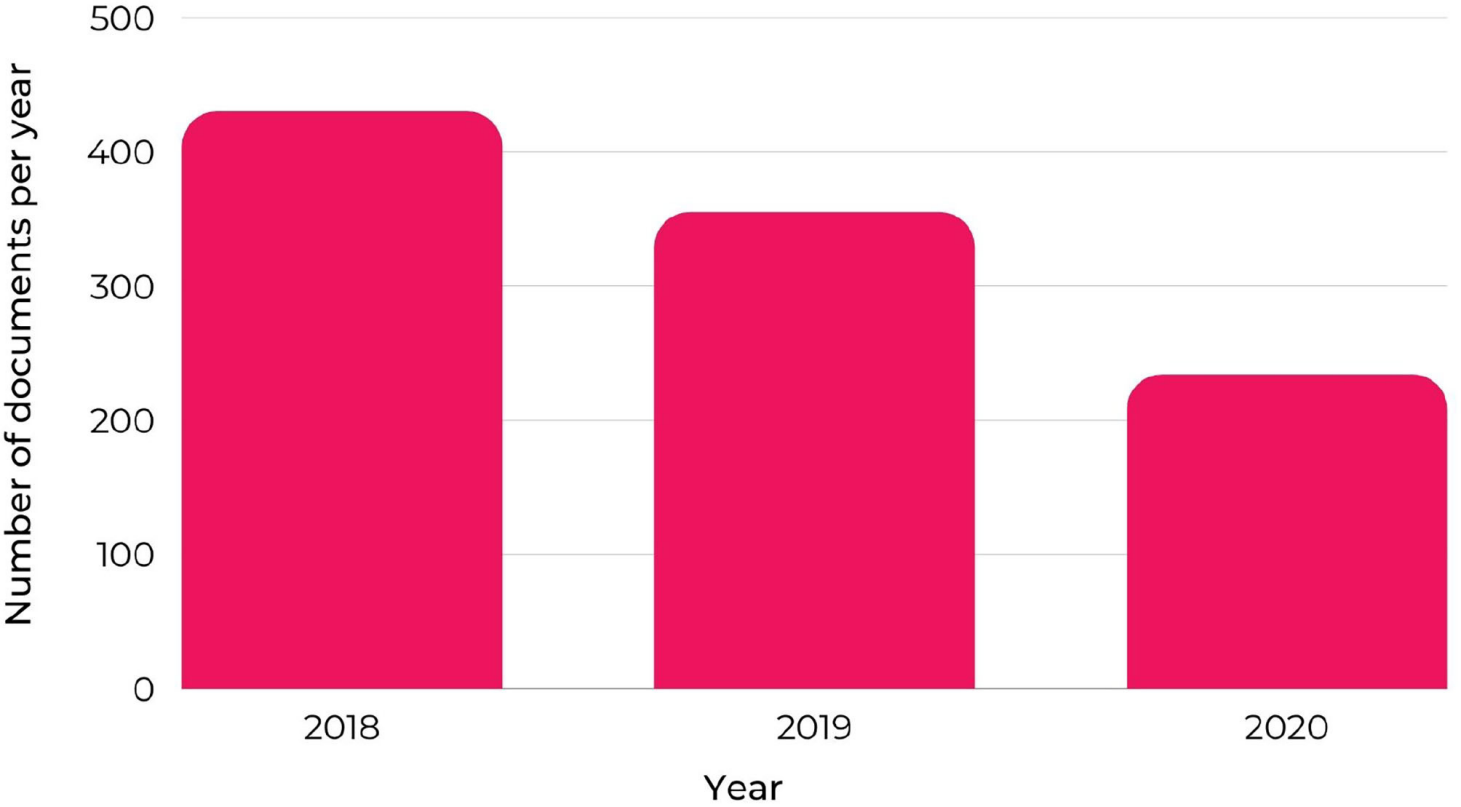
Number of reports produced in Brazil per year.

The reports describe the field researchers' intervention activities in the municipalities. So, due to their importance, they were chosen as the basis, or corpus, for applying the content analysis method. It can be seen, from Figure 3, that in 2018, 430 reports were produced; in 2019, 355; and in the year 2020, 234 reports.

**Figure 4:**
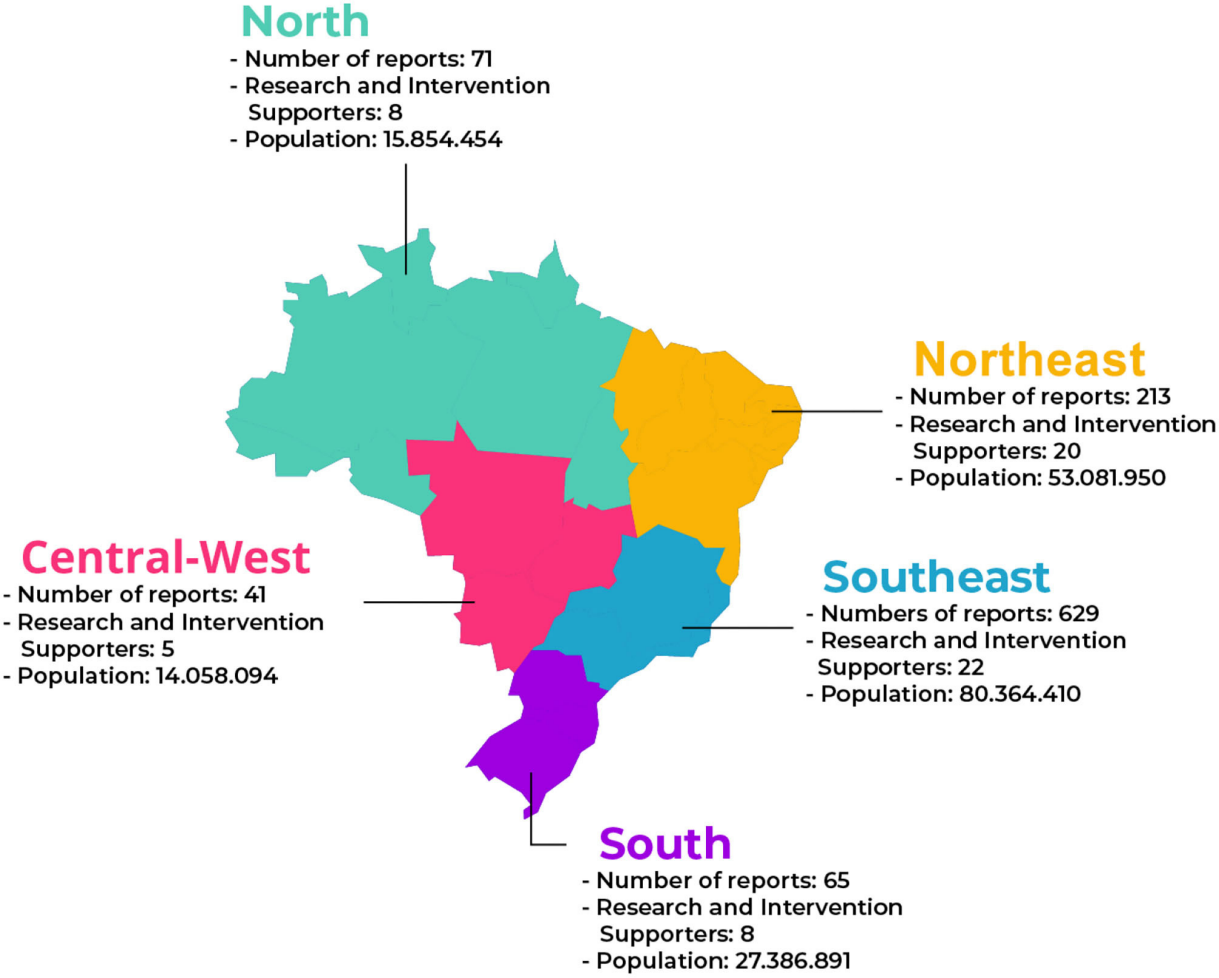
Map of Brazil with general information about the reports produced.

Figure 4 illustrates the map of Brazil with the geographical distribution of the field researchers. Besides, it describes the total number of reports produced during the three years the field researchers worked on the project and the population size by region. The Southeast of the country is the region with the most field researchers, totaling 22. According to the last census data, it is also where the higher number of reports (629) was created, being the largest region in terms of population size, with 80,364,410 people (34).

The second region that most produced reports was the Northeast, with a total of 213. It is also the second-largest in relation to field researchers (20) and population size (53,081,950). The South region produced 65 reports, and the Central-West region, 41.

### 3.1. Analysis of Bigrams

**Figure 5:**
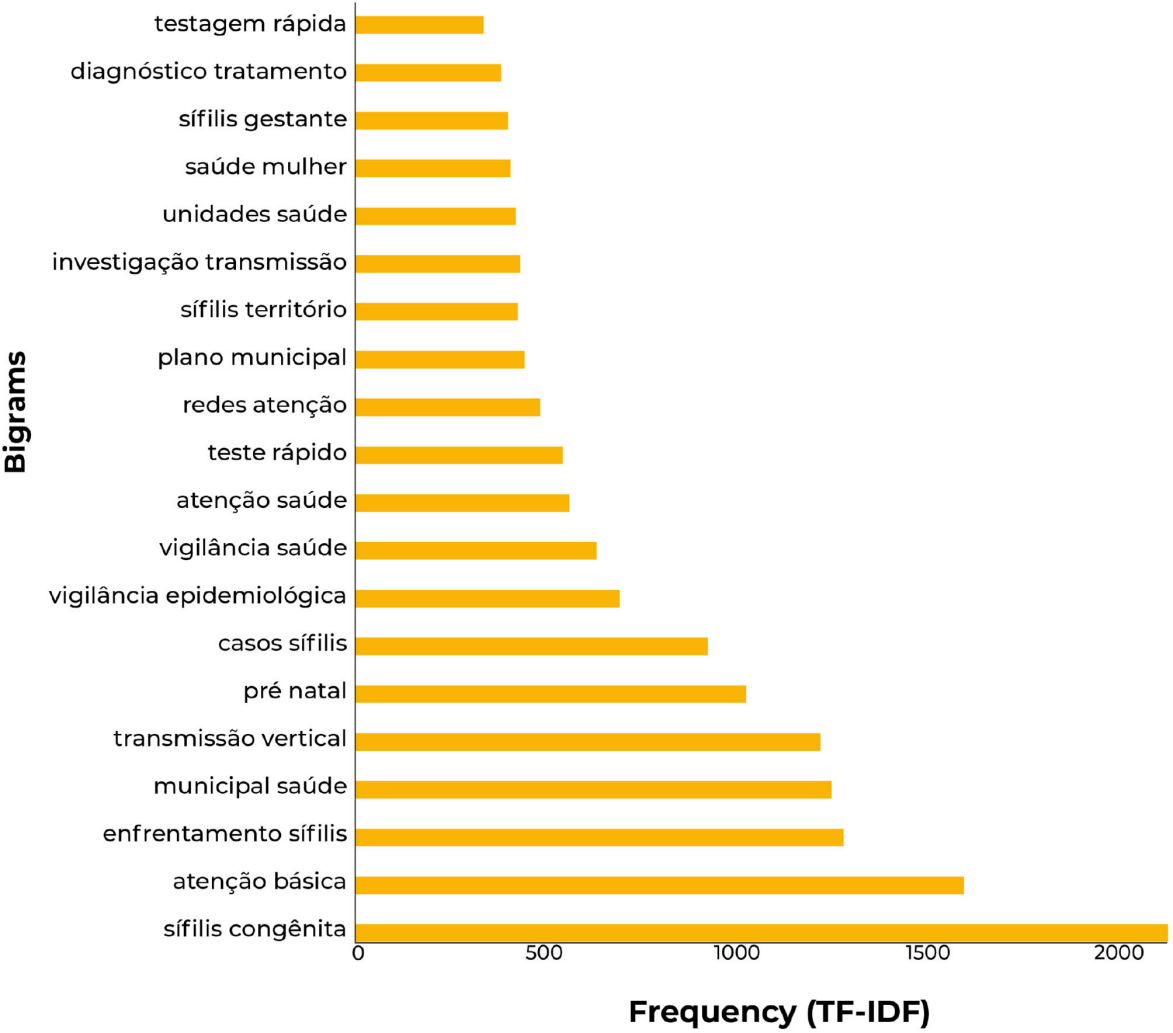
The 20 most common bigrams in reports.

The content analysis based on the bigrams has allowed the verification of which conceptual terms of “Syphilis No” present the most in the reports of the field researchers. The term “congenital syphilis” had the highest frequency. Hence, it signals that this ailment was the most important in the work reports, to the detriment of “syphilis in pregnant women,” which had a much lower frequency. Likewise, “acquired syphilis” did not figure among the 20 most present bigrams in the reports (Figure 5).

The term “primary care” was the second most frequent. It indicates that this health system component was relevant the most for the supporters when it comes to “confronting syphilis” (third most frequent term). In this vein, “prenatal care” was the most relevant health service, “women's health” was the most significant programmatic area, and “pregnant women” was the most mentioned target audience. Among the other terms, “vertical transmission,” “transmission investigation,” “syphilis cases” suggest the relevance of epidemiological surveillance efforts in response to CS.

**Figure 6:**
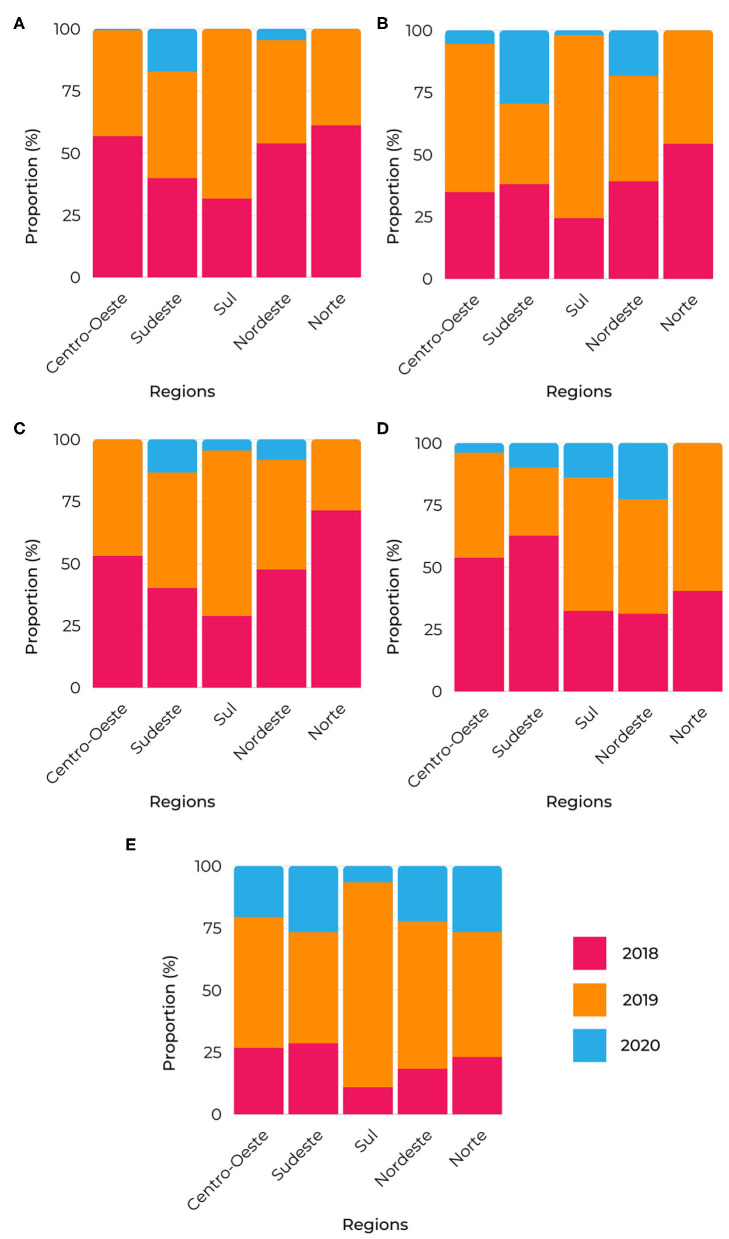
Bigrams proportion of relevant terms segmented by region. **(A)** primary care, Confronting syphilis, syphilis cases and prenatal care; **(B)** care networks, health units, women's health and pregnant syphilis; **(C)** investigation transmission, syphilis territory, and congenital syphilis; **(D)** municipal plan, epidemiological surveillance, and health surveillance; **(E)** rapid testing and diagnosis treatment.

The analysis of the bigrams allowed them to be clustered into exploratory categories related to the response to congenital syphilis and the frequency of these terms in healthcare networks, referring to the original name of the “Syphilis No!” Project. Figure 6 provides the distribution of the exploratory categories per year and region.

These are considered conceptual terms from the bigrams: “Confronting syphilis in prenatal care in primary health care” (Figure 6A); “investigation of transmission of congenital syphilis in the territory” (Figure 6B); “epidemiological surveillance within the municipal health plan” (Figure 6C); “syphilis in pregnant women in women's health care” (Figure 6D); “rapid testing, diagnosis, and treatment” (Figure 6E).

“Confronting syphilis in prenatal care in primary health care” (Figure 6A) shows that the top activities occurred in 2018 in most regions, except for the South and Southeast regions. For the “investigation of transmission of congenital syphilis in the territory” (Figure 6B), the Central-West and North regions indicated high proportions at the very beginning of the project. As for the “epidemiological surveillance within the municipal health plan” (Figure 6C), its best distribution was in 2018 in the Central-West and Southeast regions. For the “syphilis in pregnant women in women's health care” (Figure 6D), the South region exhibited the highest activity in 2019. Finally, the highest proportion of “rapid testing, diagnosis and treatment” (Figure 6E) was in 2019 in all regions, with the South region being prominent.

### 3.2. Analysis of Trigrams

**Figure 7:**
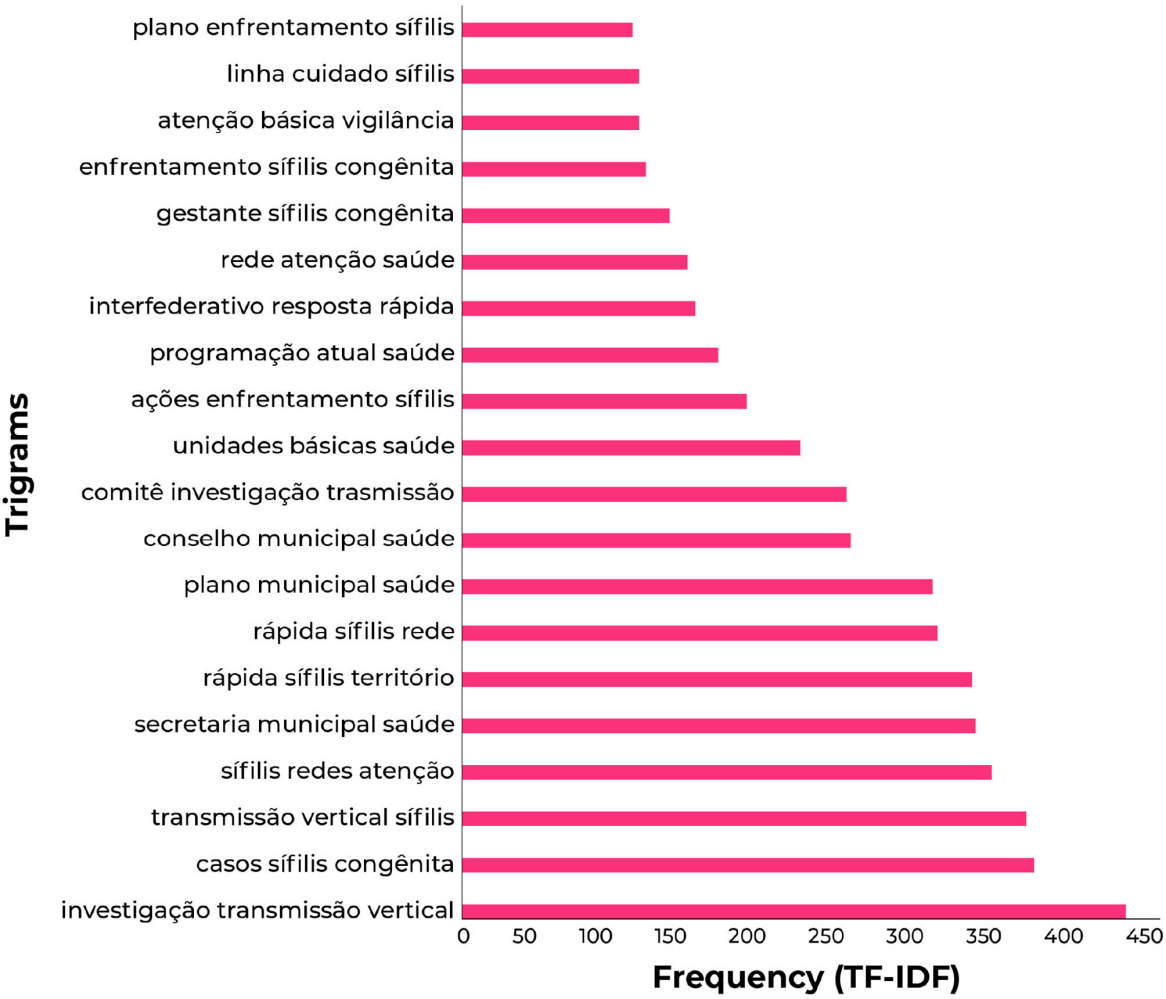
The 20 most common trigrams in reports.

The 20 most relevant trigrams extracted in the analysis have been pointed out in Figure 7, with greater relevance to “investigation vertical transmission,” “congenital syphilis cases,” “vertical transmission syphilis,” and “syphilis healthcare networks.” Such trigrams reveal that the response to MTCT (vertical transmission) of congenital syphilis–including the investigation of transmission within healthcare networks–was considered a priority in the reports.

**Figure 8:**
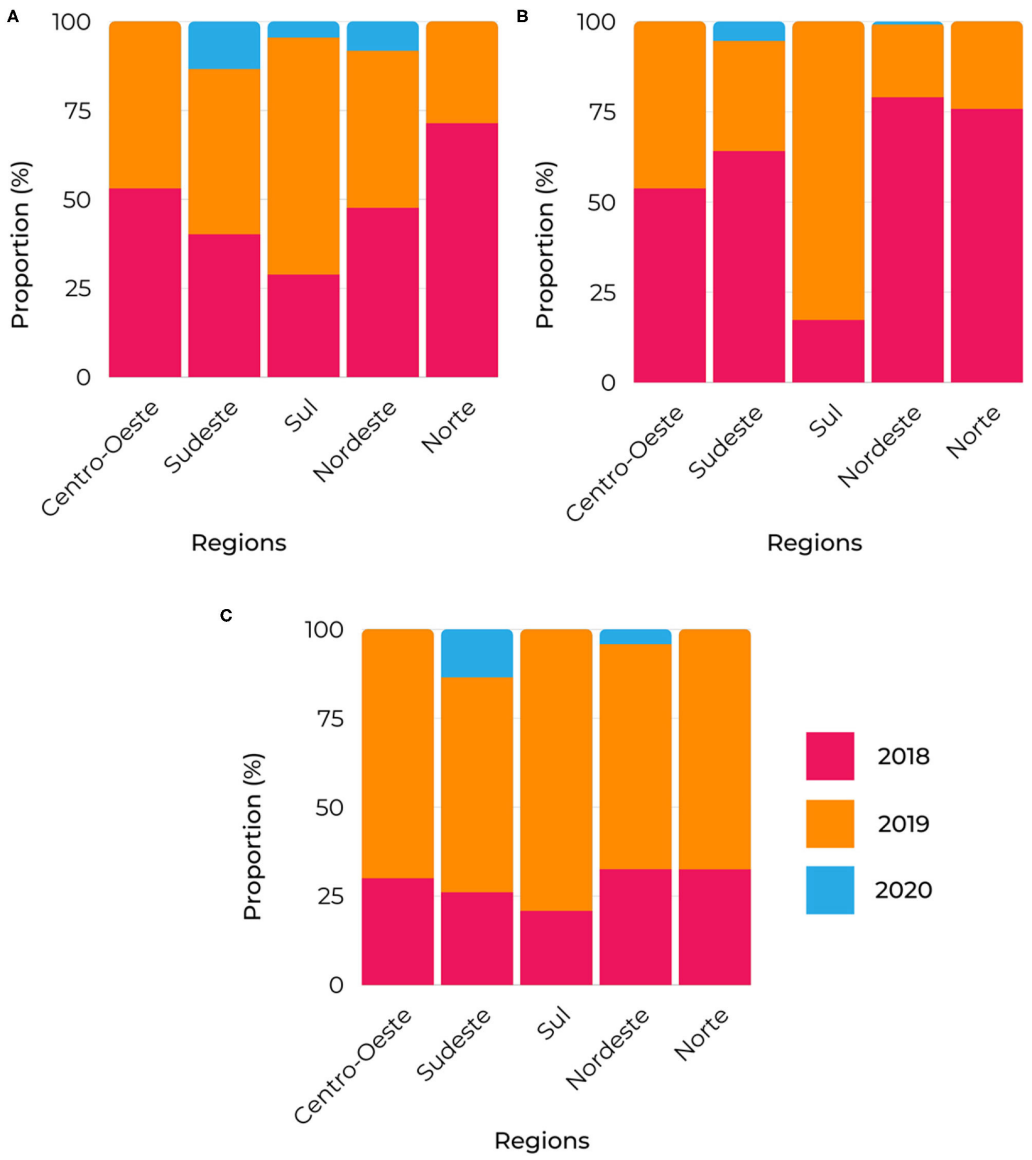
Trigrams proportion of relevant terms segmented by region. **(A)** investigation vertical transmission, congenital syphilis cases, vertical transmission syphilis and investigation committee transmission; **(B)** municipal health plan, municipal health secretariat, municipal health council, annual health program and confrontation congenital syphilis; **(C)** primary health center, rapid syphilis network, rapid syphilis territory, congenital syphilis pregnant, health care network, primary care surveillance and line care syphilis.

These are considered conceptual terms from the trigrams: “Committee for investigation of MTCT of congenital syphilis” (Figure 8A), “congenital syphilis in municipalities' planning and yearly health programming” (Figure 8B), and “surveillance and attention in the lines of care for congenital syphilis and syphilis in pregnant women in primary centers of healthcare networks” (Figure 8C).

The analysis based on the trigram clusters corroborates the tendency of the work of the supporters within the scope of the “investigation of vertical transmission” of “congenital syphilis” (Figures 8A–C). Further, “pregnant women” remains as the most crucial target population (Figure 8C), and the “municipal health plan” emerges as a relevant instrument, adding to it the “yearly health programming” and the “municipal health councils.”

Also, in Figure 8, the most significant activities for the “Committee for investigation of vertical transmission of congenital syphilis” (Figure 8A) and for “congenital syphilis in municipalities' planning and yearly health programming” (Figure 8B) occurred in 2018 for virtually all regions. Lastly, the topmost activity of the conceptual term “surveillance and attention in the lines of care for congenital syphilis and syphilis in pregnant women in primary centers of healthcare networks” (Figure 8C) was in 2019 across all the regions.

### 3.3. Analysis of Quadrigrams

**Figure 9:**
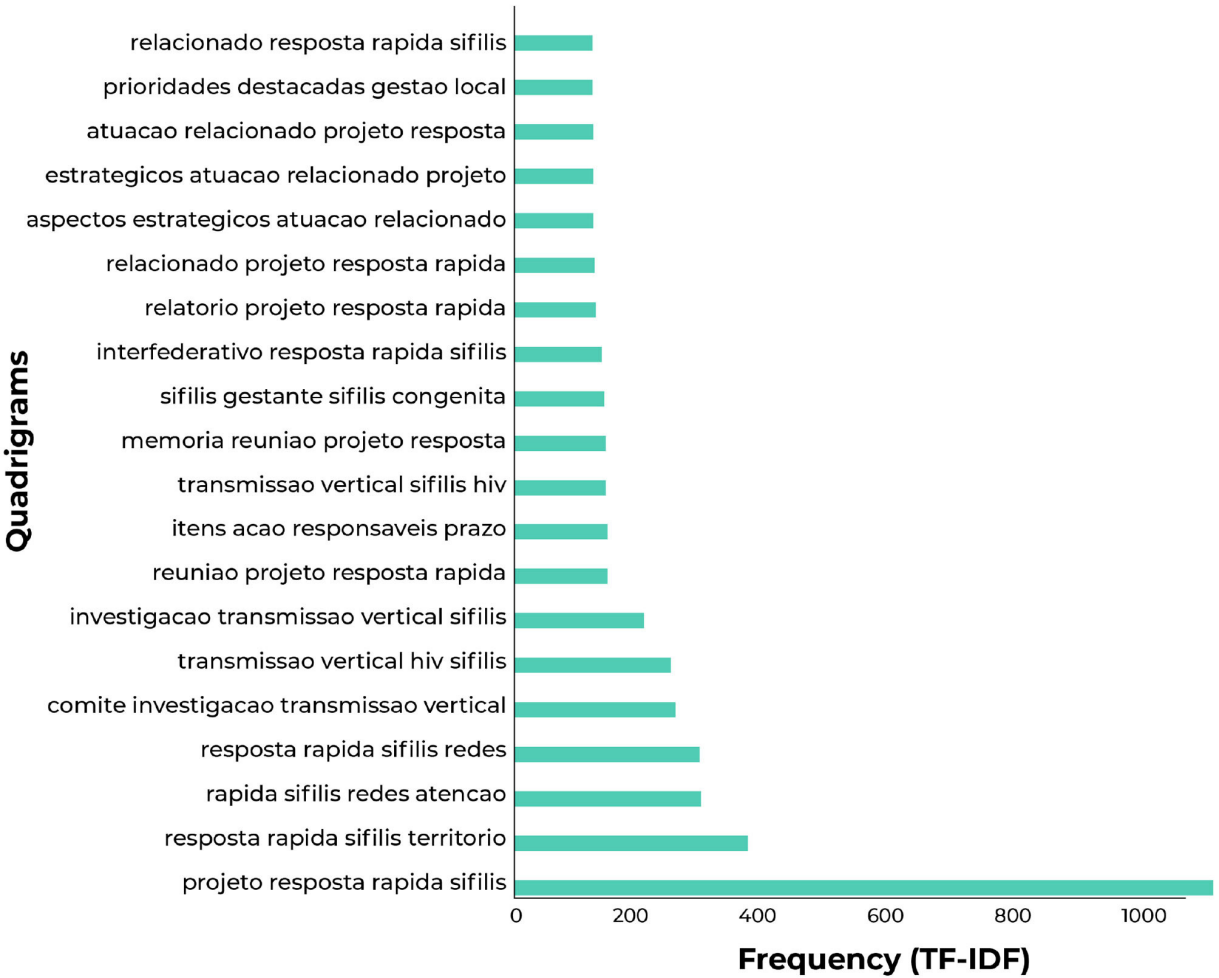
The 20 most common quadrigrams in reports.

The extracted quadrigrams can be seen in Figure 9. The highest frequency is for part of the full name of the “Syphilis No” project (“rapid response syphilis project”).

**Figure 10:**
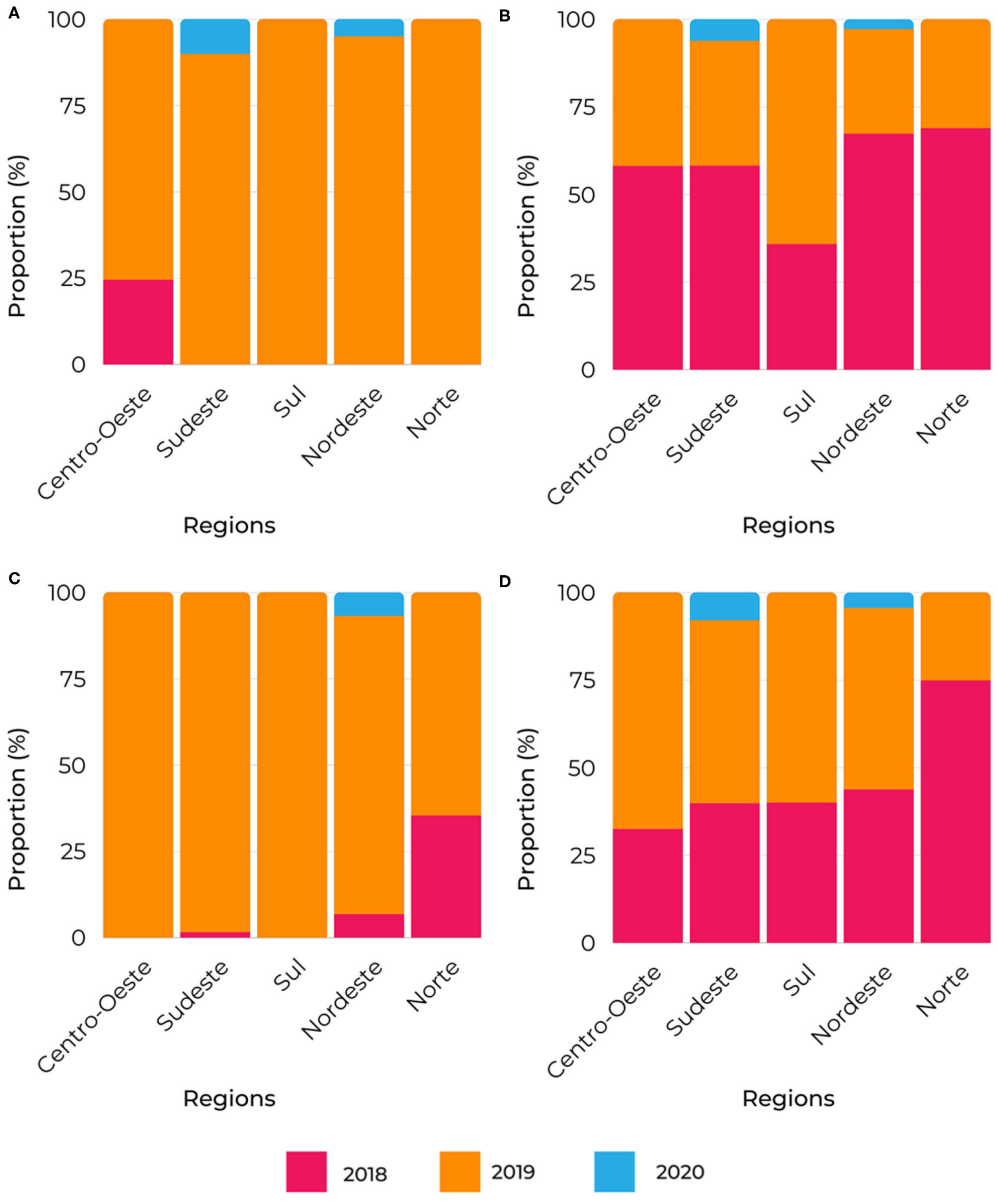
Quadrigrams proportion of relevant terms segmented by region. **(A)** rapid response project implementation, supporter related response project, main activities developed territory, strategic aspects action supporter; **(B)** rapid syphilis care networks, congenital syphilis lower year, advances rapid response syphilis, related rapid response project; **(C)** highlighted priorities local management, highlighted local management related, syphilis pregnant congenital syphilis, national day against syphilis; **(D)** investigation vertical transmission committee, investigation vertical syphilis transmission, related rapid response syphilis.

The remaining quadrigrams confirm the field researchers' priority for investigating vertical transmission and the committee as a substantial means or service for response to CS in the territory. Besides, four conceptual terms were generated and distributed according to year and region (see Figure 10).

In Figure 10A, the concept of “implementation of the rapid response project through strategic actions of the research and intervention supporter” has prevailed. As a result, this term attained its most outstanding distribution in all regions in 2019. The second conceptual term spawned was “rapid response to syphilis leads to advancements in healthcare networks regarding congenital syphilis,” with predominant distribution in almost all regions at the project's onset (Figure 10B).

In Figure 10C, the conceptual term was “local management priorities for combating syphilis in pregnant women and congenital syphilis,” with its almost total distribution in 2019 in virtually all regions. Last, the conceptual term “committee for the investigation of vertical transmission led by syphilis rapid response” had its highest distribution in almost all regions at the beginning of the project (Figure 10D).

It was noted that the quadrigrams have further underscored the possibilities for correlations between the activities of field researchers related to CS and the dimensions of Governance and Management, Surveillance and Comprehensive Care, of the SNP.

**Figure 11:**
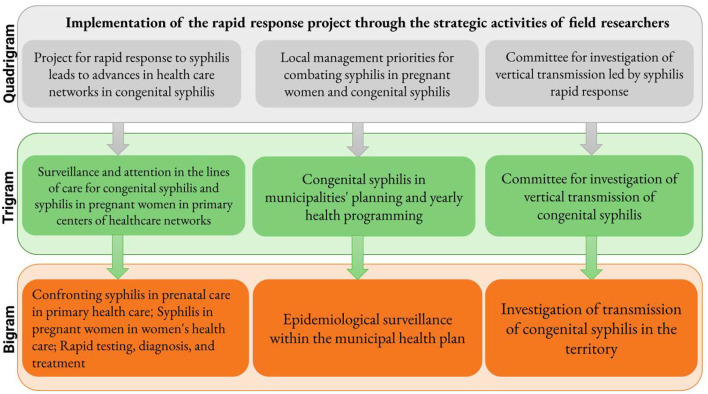
Flow diagram of literature search.

Figure 11 presents the interrelatedness between the conceptual terms formed in light of the objectives of the “Syphilis No” Project for the bigrams, trigrams, and quadrigrams. Still, the interpretation of these establishes at least five analytical categories that are considered representative of the associations between the research supporter's intervention work and the goals of the project:

1. Confronting syphilis during prenatal care in primary health care;
2. Committee for investigation of congenital syphilis in the territory;
3. Municipal plan for monitoring and investigating syphilis cases through health surveillance;
4. Women's health care networks for addressing syphilis in pregnant;
5. Diagnosis and treatment with a focus on rapid testing.

Thus, it can be inferred that the work of SNP field researchers was primarily that of intervention for confronting the response to congenital syphilis in the territory where they worked, following the line for eradication of CS by each municipality.

## 4. Discussion

The research has evidenced that using the text mining technique has enabled the identification of patterns related to the action or intervention of the field researchers' work regarding syphilis in the priority municipalities. Likewise, it has facilitated the measurement and comparison of the researchers' efforts by region of the country. Five analytical categories were determined. These demonstrate significant and representative associations between the articulation work of the Research and Intervention Supporter and the objectives of the “Syphilis No!” Project. Specifically, the categories were:

- Confronting syphilis during prenatal care in primary health care;
- Investigation committee for congenital syphilis in the territory;
- Municipal plan for monitoring and investigating syphilis cases through health surveillance;
- Women's healthcare networks for syphilis in pregnant;
- Diagnosis and treatment with a focus on rapid testing.

It is worth remarking that the field researchers' performance in the project has emerged to intervene and help the technical teams and local managers. So, one of the main actions was to reduce the high rates of congenital syphilis in priority municipalities (35). For instance, in Brazil, from 2010 to 2017, the rate of CS was from 2.0 cases per 1,000 live births to 8.8 per 1,000 live births, respectively. Thus, an increase of 338%. There was a significant upward trend in congenital syphilis in the same interval, with an AAPC of 15.75%, with no prospect of a change in the pattern (8).

In 2018, CS rates hit an all-time high, reaching the staggering incidence of 9 cases per 1,000 live births. Nevertheless, according to the most recent epidemiological bulletin, after the beginning of the SNP, the congenital syphilis rates have decreased to 8.2 cases per 1000 live births (36). As a result, there was a change in the growth trend. It went from 15.75% to a reduction of -8.8% in the annual incidence rate of CS, with a prospect for reduction over the next few years. It may reach the 90% reduction target established by PAHO in almost four years (37). In a short span, the nationwide decrease in congenital syphilis rates can be credited to the “Syphilis No” intervention model. In this manner, the epidemiological impact proved to be effective, especially from identifying the thematic areas of the intervention included in the reports of the RIS.

The research done by Pinto and colleagues assessed the impact of actions developed before and after the SNP in Brazilian municipalities based on interrupted time series analysis. These scholars have concluded that the intervention project model contributed significantly to CS rates decrease in priority areas. The conceptual terms built from the researchers' reports can point to the most significant actions that resulted in the reduced CS rates in the intervention territories.

Our findings made it possible to identify a tendency to terms clustering directed to the programmatic actions related to congenital syphilis in the priority municipalities. The trigrams and quadrigrams have solidified this trend observed in the bigrams. In addition, they went beyond with information about the work of the supporters per axis of the project.

The Pan American Health Organization's (PAHO) Framework for Elimination of Mother-to-Child Transmission of STIs in the Americas has as one of the conceptual framework's dimensions to integrate policies targeted at women and children with the related health services. That includes the prevention stages that precede pregnancy and the health of women and children after perinatal care and childbirth (37). The results of this article have evidenced that the integration between surveillance and care is part of the field researchers' work. It is corroborated by the presence of the investigation committees of MTCT. Thus, showing that the response to syphilis in Brazil draws on a strategy not foreseen in PAHO's EMTCT Plus, and therefore is innovative. Furthermore, the data reveal that the principal intervention of the supporters was in the committees' efforts. It also indicates the strategic importance of such surveillance and comprehensive care policy to eliminate CS.

The Brazilian health system has its specific protocols for investigating and intervening in identified cases of CS. This work is conducted by the investigation committees of vertical transmission or multi-professional teams of specialists. These were formed to map the problems related to mother-to-child transmission and propose solutions based on a pre-established investigation protocol to mitigate CS. The committees are integrated into SUS as spaces for technical, confidential, non-coercive, and non-punitive activities and may be linked to municipal or state health management (38, 39).

Research findings also demonstrated that these research committees played a substantial role in the “Syphilis No!” Project, cited mainly in 2018, with prominence in the Northern region. In addition, the investigation of MTCT, shown in the bigram, had its different distribution, registering the highest proportions for the Northeast and Southeast regions, keeping these activities practically constant in the 2018-2020 time frame.

Such regional discrepancies can be attributed to the organizational complexity of Brazilian healthcare networks (40), whose implementation of committees for investigation of vertical transmission will depend on each local government in a decentralized approach. Therefore, the regions with weaker consolidation of the committees have probably required more in-depth attention from the field researchers at the beginning of the project, as observed in the Northern region (41). This fact may have influenced the effectiveness of the efforts to tackle CS in that region.

The process of mining the reports of field researchers' intervention activities has also highlighted the actions to confront syphilis in prenatal care, primary health care, and syphilis in pregnant women in women's health care. In Brazil, for example, among the experiences of the Ministry of Health considered specific to the prevention of mother-to-child transmission (PMTCT) that preceded the SNP' were the project “Nascer Maternidades” (2002) and the Operational Plan for Reducing HIV and Syphilis (2007).

The project “Nascer Maternidades” aim was to ensure the identification of the pregnant woman's serological status for HIV infection during labor (42). Moreover, the Operational Plan for the Reduction of HIV and Syphilis aimed at expanding the coverage of HIV and syphilis testing in prenatal care and guaranteeing actions agreed upon with the states and municipalities since the 1990s by the National HIV/Aids Program. Such actions were strongly related to improving HIV/AIDS indicators, having as primary strategies the acquisition and distribution of antiretroviral drug supplies; infant formula for breastfeeding HIV-exposed children; and determining the routine management of HIV during pregnancy, labor and delivery, and postpartum (43).

These policies were induced through purchasing the principal supplies and the accountability for their use by the three federative entities of SUS. Therefore, it can be concluded that these strategies, on the one hand, have contributed to PMTCT of HIV. However, on the other hand, they have not been able to curb the progression of the syphilis epidemic in Brazil, according to epidemiological indicators before the project's start (36).

Hence, the SNP is probably more advanced than previous projects because it introduced a proposal to induce public health policies in an integrated and cooperative way between surveillance and care as to syphilis in the territory—having RIS a pivotal role as articulating agents. Furthermore, the data suggest that these supporters have managed to align the activities and interventions of projects related to women's health that already existed in the intervention sites with the objectives of the “Syphilis No!” Project. Specifically, this research found that the most relevant interventions were observed for the target population of pregnant women in healthcare networks.

Regarding the distribution of the supporters' interventions by region, it stands out that prevention and assistance efforts were least reported in the South region at the beginning of the project. However, that distribution increased in 2019. It is worth pointing out that the same region has the highest growth trends of CS in Brazil (8). This fact may have contributed to more resistance from local managers at the beginning of the project's implementation. So, it can be inferred that the field researchers from the South have experienced more difficulty, when compared to the other regions, in their insertion in the territory.

Rapid testing for syphilis was another aspect of health care highlighted in the field researchers' production. For instance, a study developed by Santos and collaborators (44) to identify factors that influenced the fight against syphilis emphasized that rapid testing plays a crucial role in fighting syphilis in primary health care. Another study, developed by Roncalli and colleagues, showed that, from 2018 to 2019, there was the highest availability of rapid testing to confront congenital syphilis in history, independent of other confounding factors.

In this way, it can be surmised that the intervention activities of the field researchers contributed to the strengthening of Point-of-care rapid testing (45, 46) measures for CS prevention, which remained nearly constant throughout the production of the reports in most regions in the 2018-2020 period.

The intervention activities of the field researchers of “Syphilis No!” Project may have also contributed to the decrease in the number of congenital syphilis hospitalizations nationwide. Besides, hospitalizations rates for treatment of CS were verified to have significantly dropped from May 2018 to December 2019 (10). Such data corroborate this study's results, whose assistance and surveillance actions were featured in the field reports' production and the formation of bigrams and trigrams.

Some limitations can be identified in this study. One example is that the formation of bigrams, trigrams, and quadrigrams was mined from an algorithm that highlighted the most substantial activities of the field researchers. Consequently, that may rule out activities that have been developed with the local health services management and had some significant impact on the changes in the work processes for syphilis prevention, such as words related to the education and communication axis of the project. Nevertheless, despite such a limitation, this research distinguished which efforts were most important in contributing to the current changes in the epidemiological indicators of syphilis in Brazil.

Although field researchers provided different types of data, e.g., images and videos, these were not explored as this study focused on the data mining method for written texts. As a result, such data were removed since they were out of scope and would require computer vision and digital image processing methods for information extraction. Future research could explore TDM applied to alternative types of data.

## 5. Conclusion

The study has found that when combined with the conventional content analysis method, text mining can address public health research subjects that comprise large volumes of data. This computational method made it possible to extract, from the SNP, the intervention activities of field researchers. Furthermore, it subsidized inferences on how the project's strategies may have led to a drop in CS cases in the priority municipalities.

This work offers contributions permeating several fields: (i) analysis of large sets of data through TDM techniques; (ii) strengthening the prospects for using TDM in conjunction with content analysis, broadly applied in health research involving text production to support the assessment of interventionist actions by field researchers in large-scale national projects; and (iii) evidence that efforts toward articulating and promoting public health policies in the territory have had positive effects in managing the response to congenital syphilis. In addition, the text mining of the reports pointed out there is an articulation among the actions of the field researchers regarding CS in three out of the four dimensions of the project aixes which are governance and management; surveillance and comprehensive care.

Another highlight was that the work of the field researchers revealed the links between the project's objectives and the confrontation of syphilis in the territory. Hence, it demonstrated through analytical categories how the field researchers' priority action was developed and their contribution to reducing indicators of CS in Brazil. Therefore, it is possible to infer that the field researchers' activities, highlighted in the reports, may have induced public health policies in the territory by supporting prevention and health promotion efforts to combat CS as a priority by local health services managers. Hopefully, further work will determine which field researchers' actions have positively influenced other essential indicators in the fight against syphilis. These steps are detailed in the following sessions.

## Data Availability Statement

The datasets presented in this study can be found in online repositories. The names of the repository/repositories and accession number(s) can be found at: http://vigilanciasaude.ufrn.br/files/anonymous_reports.csv.

## Author Contributions

MR: data processing, article writing and research methodology development. RF: data processing and research methodology development. MS and TL: article writing and research methodology development. ASPM: literature review and article writing. AC-O, AEM, CO, HO, and CG: guidelines and review of the article. RP: writing and reviewing the article. DB: literature review, writing and reviewing the article. RV: article writing, review, research coordination, and research methodology development. All authors read and approved the final manuscript.

## Funding

This work was supported by Brazilian Ministry of Health–Number's project: 54/2017.

## Conflict of Interest

The authors declare that the research was conducted in the absence of any commercial or financial relationships that could be construed as a potential conflict of interest.

## Publisher's Note

All claims expressed in this article are solely those of the authors and do not necessarily represent those of their affiliated organizations, or those of the publisher, the editors and the reviewers. Any product that may be evaluated in this article, or claim that may be made by its manufacturer, is not guaranteed or endorsed by the publisher.
